# The role of forensic anthropology in disaster victim identification (DVI): recent developments and future prospects

**DOI:** 10.1080/20961790.2018.1480460

**Published:** 2018-10-02

**Authors:** Hans H. de Boer, Soren Blau, Tania Delabarde, Lucina Hackman

**Affiliations:** a Netherlands Forensic Institute, The Hague, The Netherlands;; b Department of Pathology, Academic Medical Centre, University of Amsterdam, Amsterdam, The Netherlands;; c Department of Forensic Services, Victorian Institute of Forensic Medicine, Melbourne, Victoria, Australia;; d Department of Forensic Medicine, Monash University, Monash, Australia;; e Institute of Legal Medicine, Paris, France;; f Centre for Anatomy and Human ID (CAHID), University of Dundee, Dundee, UK

**Keywords:** Forensic science, forensic anthropology, disaster victim, human identification, mass fatality, Bayes, radiology, dead body management

## Abstract

Forensic anthropological knowledge has been used in disaster victim identification (DVI) for over a century, but over the past decades, there have been a number of disaster events which have seen an increasing role for the forensic anthropologist. The experiences gained from some of the latest DVI operations have provided valuable lessons that have had an effect on the role and perceived value of the forensic anthropologist as part of the team managing the DVI process. This paper provides an overview of the ways in which forensic anthropologists may contribute to DVI with emphasis on how recent experiences and developments in forensic anthropology have augmented these contributions. Consequently, this paper reviews the value of forensic anthropological expertise at the disaster scene and in the mortuary, and discusses the way in which forensic anthropologists may use imaging in DVI efforts. Tissue-sampling strategies for DNA analysis, especially in the case of disasters with a large amount of fragmented remains, are also discussed. Additionally, consideration is given to the identification of survivors; the statistical basis of identification; the challenges related to some specific disaster scenarios; and education and training. Although forensic anthropologists can play a valuable role in different phases of a DVI operation, they never practice in isolation. The DVI process requires a multidisciplinary approach and, therefore, has a close collaboration with a range of forensic specialists.

## Introduction

A disaster has been defined as “a serious disruption of the functioning of a community or a society causing widespread human, material, economic and/or environmental losses which exceed the ability of the affected community or society to cope” [1]. While a disaster may be natural or human induced, few countries escape events which result in multiple fatalities [2, 3]. Identification of the victims of these events is considered an important mark of respect not only for the deceased but also for surviving family and friends. In addition, identification may be required legally, for instance to aid criminal proceedings, facilitate settlement of estate and/or inheritance, or the right of the remaining partner to re-marry. Consequently, specific processes have been developed to facilitate positive identification of the deceased [4].

The minimum number of fatalities that constitutes a “mass disaster” differs between jurisdictions, varying between two [5] and 10 deceased (2016, personal communication with Leditschke; unreferenced). In order to formalize the identification process following a mass disaster, the International Criminal Police Organization (INTERPOL) developed specific guidelines and protocols for disaster victim identification (DVI) which involve the collection and comparison of antemortem (AM) and postmortem (PM) data. INTERPOL has 190 member countries and, while the guidelines are not compulsory, they are recognised globally [6].

The DVI process has had broad coverage in the literature. This has included detailed descriptions of the five phases that cover the time directly following the disaster up to the burial/cremation of the deceased [4]:

- Phase 1: the disaster scene
- Phase 2: the mortuary/PM data collection
- Phase 3: AM data collection
- Phase 4: reconciliation
- Phase 5: debrief

In addition, the literature has covered the need for detailed AM data [7, 8]; establishing temporary mortuaries to deal with DVI [9, 10]; methods of packaging and preserving remains at the scene [11]; the development of quantitative decision support tools [12, 13]; country specific approaches to DVI [14, 15] together with numerous DVI case studies [16]; the politics associated with DVI [17]; the need for cultural sensitivity towards those victims and families [18, 19]; preparation and training exercises [4, 20, 21], as well as the roles of various forensic specialists involved in DVI including the forensic pathologists [22], forensic odontologists [23–25], molecular biologists [26–29], forensic radiologists [30–33], and relatively recently, the forensic anthropologists [34, 35].

Forensic anthropological knowledge has been used in DVI for over a century [34], but it was not until 1970 that the American anthropologist Thomas Dale Stewart emphasised the value of including forensic anthropology in the identification process [36]. Since this time, there have been a number of disaster events which have seen an increasing role for the forensic anthropologist in DVI. This increasing role has been augmented by feedback given after the 2004 Boxing Day Tsunami in which it was recognised that the presence of a forensic anthropologist could have been useful in many occasions [37, 38]. The lack of forensic anthropology protocols with INTERPOL and the limited possibility to enter physical anthropology data into the used case management system (PLASS DATA) added to issues with the use of this specialty. Recognition of the value of the role of forensic anthropology in the DVI process has been reflected in the inclusion of forensic anthropologists in the INTERPOL Pathology and Anthropology Sub-Working Group (PASWG) [39]. This sub-working group has provided a document to INTERPOL detailing the roles and responsibilities of the forensic anthropologist for DVI which will be included in the next version of the INTERPOL DVI guide.

While the forensic anthropologist plays a role in different phases of a DVI, they do not practice in isolation [40]. Forensic anthropologists work as part of a team of forensic specialists, which typically includes forensic pathologists, forensic odontologists, radiologists, fingerprint examiners, molecular biologists, mortuary technicians, and photographers. The specific role of the forensic anthropologist in each of the five phases of the DVI operation will be determined by the condition and preservation of the deceased persons and the context and the scale of the disaster [41, 42].

There are multiple types of disasters of varying scales which may occur naturally (e.g. hurricanes, tsunamis, bushfires, and house fires), or be human induced (e.g. aviation, train and vehicle accidents, sieges and terrorist attacks). Further, disasters may be described as “open” (when the exact number of deceased individuals is unknown at the time of the incident, such as a terrorist bombing of a building), or “closed” (when a list of the decedents exists, such as generally in aviation accidents) [4]. Regardless of the type or scale, disasters involve a range of forces that impact on the body, potentially resulting in varying forms of preservation. These may include (but are not limited to) intact or near complete bodies; recognisable body parts; soft tissue masses; isolated complete or traumatised bones (with or without associated different degrees of burnt and/or decomposed soft tissue); small un-diagnostic bone fragments; or a combination of these.

The aim of this paper is to provide an overview of the way in which forensic anthropologists may contribute to a DVI operation, with emphasis on how the experiences gained from some of the latest DVI operations and the recent developments in forensic anthropology impact the various aspects of the DVI process.

## At the disaster site

At the disaster site, the pressure to locate and collect remains to facilitate timely identifications generally competes against a background of chaos and limited resources. In such challenging environments, experience has shown that the detailed mapping and recording of bodies, body parts, bones (complete or fragmented) and associated evidence is of vital importance [43–45]. As Hinkes [46] highlighted nearly 30 years ago, the ability to recognise fragmented and otherwise compromised remains is vital in a DVI situation [34]. It is axiomatic to state that if human remains, regardless of their preservation, cannot be recognised at the scene then they cannot be recorded and appropriately collected.

The initial evaluation of the condition and preservation of the remains at the scene significantly impacts on planning logistics for complete recording and recovery of human remains and, thereafter, the subsequent stages of the DVI processes [34]. Timely management of the scene is important to prevent further unnecessary fragmentation or decomposition. Based on their expertise in dealing with differentially preserved remains, forensic anthropologists can make a critical contribution at the disaster site. Their assistance at the scene will help to prevent the collection of items such as non-human remains or non-osseous items, thereby reducing the allocation of case numbers and the generation of superfluous data [45]. In addition, their assistance at the scene ensures that *all* the body parts/fragments have been collected, thus minimizing the necessity to re-examine the scene. Lastly, when the bodies are compromised, they can advise on the best means of packaging and transporting the remains in order to minimize damage in transit.

The added value of forensic anthropological expertise at the scene is illustrated by several examples. Following the 2001 terrorist attack on the World Trade Towers in New York, fire fighters predominantly undertook the initial recovery process. As they were not trained in forensic anthropological/archaeological techniques and had no experience recognizing heavily fragmented and disrupted human remains, the recovery efforts resulted in additional commingling which in turn complicated and slowed identifications [47]. Similarly, following the 2009 bushfires that affected the state of Victoria, Australia, initial examination of many of the scenes did not include a forensic anthropologist (predominantly because of the limited numbers of forensic anthropologists). This meant that scenes had to be searched more than once which had time and financial implications for the identification process [48]. More recently, after the MH17 airplane incident in Ukraine in 2014, local volunteers predominantly undertook the initial recovery process. This was unavoidable given the backdrop of an on-going civil war, but it complicated and slowed the subsequent identification process.

The mapping of the disaster site is generally not the primary concern of the forensic anthropologist. However, in many countries, forensic archaeologists and forensic anthropologists work closely together and sometimes practitioners have both anthropological and archaeological skills [49]. As such, the developments in forensic archaeology in mapping, searching, and processing a crime scene or disaster site have a direct effect on the skills that the forensic anthropologist can bring to the scenario.

The INTERPOL DVI guide advocates the use of a gridding system to map a disaster scene and the use of printed recovery labels to label all the bodies or body parts found at the scene [50]. This methodology has its merits, especially in low-tech environments, but over the past years more advanced methodologies have been developed [43, 44, 51, 52]. The use of electronic mapping equipment such as total stations, drones, or hand-held GPS devices have become mainstream in forensic archaeology [51, 53] and their (combined) use enables a DVI team to quickly map a disaster site. The incorporation of the thus acquired mapping in a Geographical Information System (GIS) will provide important information, not only for planning purposes but also to subsequently record the location of the human remains. For the latter, the use of hand-held devices (such as mobile phones or other GPS-linked devices) can be used to electronically record the location of a body part or other types of evidence. Amongst others, this will result in an automatically compiled list of recovered items. A direct link with the DVI database such as DVI System (by PLASS DATA) limits the administrational burden.

These more advanced techniques will prove particularly useful in cases of large and complex disasters sites, and at disaster sites where human remains and other types of forensic evidence are simultaneously recovered.

## In the mortuary

Over the past years, forensic anthropologists have assisted in the investigation of mass disasters by undertaking a range of analyses, including:

- separating osseous from non-osseous material;
- confirming that the remains are human (or non-human – if not done at the scene [45]);
- separating recognizable versus non-recognizable fragments that require DNA analysis;
- identifying and managing commingled remains [54, 55] (which may involve re-associating disparate body parts [56]);
- providing a biological profile (an estimation of the person’s ancestry, sex, age, and stature), if possible including other identifying information such as previous fractures, disease, or anatomical variants;
- assisting in reconstructing the manner of death, for instance in case of bullet trajectories or locating shrapnel.

In some cases, examinations in the mortuary inform how new scene examinations are to be undertaken. For example, after an initial examination of the disrupted bodies of terrorists from the Paris November 2015 attacks, a second recovery phase was performed in the Bataclan concert hall to locate missing body parts.

In many DVI contexts, identification will be confirmed relatively quickly through odontology, fingerprints or DNA [57]. However, there are many reasons why these methods may be delayed or in some cases, impossible to implement. The preservation of the body (part) (e.g. due to skeletisation, fragmentation and/or degradation), and the quality, quantity and availability of antemortem data can all limit the utility of aforementioned methods. It is well-recognized, for example, that marginalized communities are often those most susceptible to mass fatalities and are also those that are the least likely to have antemortem records such as dental charts and X-rays. The development of a biological profile at the triage stage may, therefore, provide a helpful “snapshot’ of the identity of the person before antemortem information is located. This may provide important leads for a positive identification and thus expedite identification. Typically, the most useful biological parameters in this regard are the estimation of sex and age at death, the utility of ancestry and stature is generally of limited value [58]. Other potentially useful information that can be provided by the forensic anthropologist are details about skeletal pathologies [57] and skeletal anomalies and variation [59].

It is now well recognized that population-specific standards are required when developing a biological profile. For this reason, large numbers of research projects are being undertaken to develop objective and standardized anthropological methods, or to test the accuracy of methods outside the population they were derived from. On-going research continues to augment the contributions of forensic anthropology to human identification [60]. The degree to which these can be employed will naturally depend on the context and the nature of a disaster.

### Imaging techniques

Imaging methods such as radiographs and postmortem computed tomography (PMCT) scans are increasingly used during DVI operations, notably due to the emergence of portable X-ray machinery and mobile CT scanners. The analysis undertaken in the mortuary by the forensic anthropologist is, therefore, increasingly likely to involve the analysis of such radiological images [61–64].

The use of radiological imaging has been proven to be beneficial to the identification process in multiple ways. It may assist in identifying and re-associating body parts [65], as well as documenting information that could be used for identification, such as the presence of individualising features [66], dental restorations [67], surgical implants/interventions, evidence of (partially healed) bone trauma, and personal artefacts [30, 64, 68]. In addition, when available, AM scans can be compared with the PM scans in order to provide a (tentative) identification. Anatomical traits that could be used for this purpose include the morphology of the paranasal sinuses [66] or the vascular grooves on the endosteal surface of the cranium [69].

The use of PMCT scanning is also useful to give a quick overview of body bag contents and provides an easy way to record the received remains in their “*in situ*” state. This is especially helpful when operatives of the DVI team did not perform the recovery. The scans can furthermore be helpful when the victims are not autopsied in full, for instance for documentation or re-examination purposes [70–72].

Imaging may also be used by forensic anthropologists for the development of varying aspects of the biological profile of a deceased person. Over the past years, there has been a significant increase in the amount of research undertaken which combines radiological imaging techniques with forensic anthropological methods [73–75]. To date, metric forensic anthropological techniques cannot be readily applied on volume rendered 3D reconstructions because there is little knowledge about how the accuracy of the method is affected by the use of digital images, for instance through landmark recognition or observer variability [76]. This limitation does not seem to hold for the ordinary planar reconstructions that can give comparable results compared to measurements of the original osteological material. Research has demonstrated that the results obtained when using some morphological forensic anthropological methods are comparable to the result from the same methods performed using CT scans [77], but more research is still needed.

In many cases, the work involving the use of imaging may overlap with the forensic radiologists [62, 72, 78] and forensic odontologists [79]. It is, therefore, imperative that forensic anthropologists liaise closely with these colleagues.

### DNA sampling and the handling of fragmented remains

Where DNA is required for the identification process, forensic anthropologists (in collaboration with biologists) can contribute to the development of DNA sampling protocols [80, 81]. In cases of heavily disrupted human remains which typically result from bombings or airplane crashes, forensic anthropologists can contribute substantially by using their knowledge of bone biology and taphonomy to select the most appropriate samples for DNA analysis [82–84]. For example, during the DVI operation following the 2002 Bali bombing, which relied heavily on DNA [85], the retrieval and identification of appropriate soft tissue and bone fragments for DNA testing was paramount. Because a high degree of fragmentation is typical of individuals close to a blast site [86], the ability to recognise highly fragmentary remains was also important in providing details about individuals thought to be at the epicentre of the explosion [87].

Depending on the nature of the disaster, the scale of fragmentation and commingling may require a management plan dealing specifically with fragmentary remains [88]. If the decision is made to re-associate every body part with a named individual, the definition of what constitutes a body part must be clearly stated and communicated to scene and mortuary personnel. Currently, there is no standard definition of a “body part” and definitions have included: all suspected human tissue greater than 5 cm× 5 cm; human tissue containing at least 5 cm of bone; human tissue with “a fair chance of identification”; and only those parts that can be anatomically identified, regardless of the size.

Following the MH17 airplane incident in 2014, the majority of the recovered human remains were typical of an aviation disaster, that is, extensively commingled, skeletonised and/or fragmented. During the DVI operation it was eventually decided that every non-calcined human bone fragment weighing more than 3 g that could not be re-associated with another skeletal fragment would be submitted for DNA-analysis. While the exclusion of non-human material and the re-association of larger human skeletal fragments by the forensic anthropologist considerably reduced the number of samples submitted for DNA, nonetheless thousands of bone fragments required analyses (both morphological and DNA). This illustrates the need for the effective management of body parts with proactive strategic planning and managerial decisions when a large number of fragmentary remains are recovered. Experience has shown that these issues should be addressed as early as possible, preferably in the initial strategic planning stages of the DVI operation. Input by forensic experts, including forensic anthropologists, is imperative to finalising a prudent working plan for each particular context. While some organizations such as the Asia Pacific Medico Legal Agencies (APMLA) have produced documents related to the management of fragmentary human remains [88], the INTERPOL PASWG is currently working on developing a document that will facilitate the identification of the most important managerial decisions and augment the recording of skeletal/fragmented remains.

## Identification of the living

Although DVI generally focuses on the identification of the deceased, the identification of victims who survive a mass fatality event also needs consideration in any DVI response. Mostly the identification of the living does not require forensic anthropological expertise, but recent disasters have shown that forensic anthropologists may be included in the process [89].

Identification of the living is important in both open and closed disasters. However, the timely identification of survivors in an open disaster has an important impact on the identification process as it enables their elimination from the missing persons list. It might also impact those treating survivors, since medical teams will be confronted with the need to deliver medical treatment in absence of (medical) background information. When the survivor is an unidentified minor, the lack of consent to medical treatment from appropriate adults must also be taken into account.

Survivors belong to one of the four groups. The first group includes those who are uninjured and are thus expected to leave the site of the fatality event by themselves. The second group includes those who are injured but still conscious. The forensic anthropologist’s involvement in both groups is minimal [90]. For individuals who receive medical treatment, it should be kept in mind that any identifying data collected by the medical team will need to be forwarded to the identification team and contains the details required for identification. For this, the person in charge of the identification process should task officers to specifically recover and collate these data at medical care centres.

The final two groups include those who are injured but unconscious and survive their injuries; and those who are injured, unconscious, and succumb to their injuries in hospital. These groups present what may be perceived as the greatest issue to the identification process, constituting those individuals who are so severely injured that they are unable to communicate their details. In these cases it is becoming increasingly common to attempt identification through the application of the same processes that are used to identify the deceased. This approach proved to be hugely effective following the terrorist attacks in Paris in 2015 and Nice in 2016 [71, 91]. However, the effectiveness of this approach is dependent upon the fact that until identified, the severely injured form part of the presumed missing group for whom AM data will be collected.

In order to complete the DVI documentation for identification purposes, the same information is collected from both the unconscious patient and the deceased person [92]. DNA swabs are recovered to create a DNA profile. Fingerprints and dental status can also be recovered if possible, although the success is dependent on the injuries received [93].

For the recovery of any further information, medical imaging plays a central role and the common use of radiographs and CT scans during medical triage and treatment ensures their availability. The forensic anthropologist can complement the analysis of radiologists, forensic pathologists, and forensic odontologists, by developing a biological profile, or comment on the presence of pathologies or implants and other information which can be used to direct identification data collection and matching [58, 59, 65, 94–98]. Also knowledge on the variation in external features such as skin colour and hair colour may prove beneficial to the identification process. Identification of the living in this way can work alongside the identification of the deceased ensuring that the identification process does not stall due to lack of information.

## The use of Bayes’ theorem in forensic anthropological identification

Forensic anthropologists are increasingly (made) aware that they need to quantify the performance of their methods [99] and this has led to a subsequent increase of probabilistic statistical methods in forensic anthropology [100]. This in turn has had an important effect on the manner in which forensic anthropologists approach human identification [101–103]. Knowledge of the theoretical background of this development, and the ability to incorporate the latest statistical methods in DVI fieldwork is beneficial in order to substantiate proposed identifications and is, therefore, increasingly requested of forensic anthropologists.

Characteristically, forensic anthropologists focus on bias, precision, and accuracy of their methods. Bias and precision relate to the systematic error of a method, for example, inter- and intra-observer variation and statistical variance. The accuracy of a method is defined by the extent in which the results of the method are in concordance with the true value, for instance the percentage of correct sex estimations.

For human identification purposes, these test characteristics are preferably combined with contextual data using a Bayesian approach, and the increasing use of this approach requires forensic anthropologists to be aware of its use and premises. Bayes’ theorem describes the way one's prior beliefs about a particular event are informed or updated based on the consideration of additional evidence [104]. It is commonly used in various methods of human identification of which comparative DNA analysis is probably the most well-known example [105–107]. The theorem dictates that the *posterior odds* of an identification is provided by the multiplication of the *prior odds* of that identification with the *evidential value* of a specific observation [108]. In other words, the probability of a correct identification (the posterior odds) is as much dependent on the probability of a correct identification prior to carrying out an identification method (the prior odds) as on the evidential value of that same identification method.

This evidential value is given by the likelihood ratio. The likelihood ratio is a ratio of two probabilities, namely the probability of an observation given a proposed hypothesis is true, and the probability of the same observation given an alternative (mutually exclusive) hypothesis is true. As such, the likelihood ratio expresses the magnitude by which a specific piece of evidence affects the probability of two competing hypotheses. In the context of forensic anthropology and human identification, the likelihood ratio can be used to express to what extent an identification becomes more or less probable, given the results of a (forensic anthropological) test. As a by-effect, it can also be used as a means to predict the added value of an identification method given the context of the case. See [108] for an excellent introduction on the application of a Bayesian approach in forensic settings.

Bayes’ theorem can be used in two different scenarios that the forensic anthropologist might be confronted with [102]. In the first scenario, the forensic anthropologist compiles a biological profile of the remains in order to provide leads for identification. In the second scenario, the forensic anthropologist tests a tentative identification against the biological information of the remains.

The adoption of a Bayesian approach has multiple benefits. First, it allows a forensic anthropologist to quantify the evidential value of an observation in a transparent way. Second, it allows for quick assessment of which reference information is needed for an appropriate probabilistic statement. The gathering of such reference data is generally challenging, but it is expected that with the development of ever more sophisticated methods to quantify human variability (e.g. machine learning and automated image processing) and the increasing availability of large-scale reference data (e.g. through governmental or medical databases) our knowledge of prior odds and the evidential value of forensic anthropological methods will increase substantially. Third, the use of likelihood ratios allows for a relatively easy combination of various pieces of evidence, either forensic anthropological in nature or from other forensic disciplines.

It is important to note that the subjectivity of aspects of forensic anthropological methodologies does not preclude the calculation of a likelihood ratio. A trait does not have to be unique or scarce in order to have evidential value. Any trait has evidential value. Naturally, less subjective and more accurate methods will result in higher likelihood ratios, but even relatively low likelihood ratios may provide useful information. Forensic anthropological observations which generally provide relatively low likelihood ratios can produce considerably strong evidence for a tentative identification when combined [109]. This is especially relevant in those cases in which the so-called scientific methods with generally higher evidential values (such as DNA, fingerprints or odontology) are not feasible.

## The changing nature of DVI operations

Every disaster is unique and consequently, every DVI response is faced with different and sometimes unprecedented challenges. Despite this, particular trends in the scale and type of disasters are apparent. These trends have changed the way authorities and forensic experts think about preparing and implementing DVI [42, 110], and have highlighted some limitations associated with the traditional INTERPOL DVI process [111].

Large scale disasters – events resulting in the fatality of tens to hundreds of thousands of people – are increasingly prevalent [112]. For example, a total of 13 countries were impacted by the 2004 Boxing Day tsunami with over 226 000 fatalities [113]. The Thai tsunami victim identification (TTVI) operation was the largest victim identification operation in history. Initially 5 395 victims were recovered, of which approximately 3 308 were identified (predominantly from dental records) from 40 countries after three and a half years of investigations [85]. This operation was considered by some as one of the most successful of its kind [114], but the financial and time costs of the process have led others to describe it as a huge effort with a modest result [115]. Consequently, there has been increasing recognition of the importance of appropriate dead body management [116–118], which typically involves carefully planned mass burial [116, 117, 119, 120]. Professional dead body management is not only an initial means of respecting the deceased at a time when local infrastructure and capacity is all but destroyed, but it is also a means of augmenting the possibility of future identification. Dead body management requires increased awareness among those initially impacted by the disaster and forensic anthropologists have played a pivotal role in providing training in dead body management to these first responders [112].

Another relatively recent challenge is constituted by the huge number of deaths related to the (refugee) migrations taking place in for instance the Mediterranean region [121], Sub-Saharan Africa, at the US-Mexican border and in Australasia. In essence, each region should consider these deaths to be part of a massive, multinational, and protracted disaster that requires an equally international and intricate DVI response [122]. The general lack of a missing persons list (and consequently of AM data), and the need to integrate PM data from different countries and mortuaries, calls for an unprecedented degree of collaboration between governments, humanitarian organisations and forensic practitioners. Since the identification of deceased migrants is generally not feasible by DNA, odontology or fingerprint analysis, the development and implementation of alternative identification methods, such as forensic anthropological biological profiling is required.

Mass casualties following a terrorist attack constitute another type of disaster representing specific challenge for DVI teams. The criminal nature of such an event usually changes the priorities of the overseeing governmental institution, as authorities are confronted with the need to combine a criminal investigation with the requirement to identify the deceased. In these cases, the DVI operation is usually secondary to more urgent matters such as the search for perpetrators and/or the anticipation of further attacks. Several countries have specialized teams of first responders for terrorist incidents, and it is imperative that DVI teams are aware of their role within the wider criminal investigation. Based on recent experiences, DVI teams should be prepared for a variety of scenarios such as single or multiple disasters sites, either occurring at once or consecutively. Also, they should be prepared for different types of attacks such as shootings, stabbings, (suicide) bombings, vehicle(s) charging into people or Chemical, Biological, Radiological and Nuclear (CBRN) scenarios. A close collaboration between the criminal investigators and the DVI team, who inevitably will jointly conduct their respective investigations, ensures that victims, perpetrators, and all types of evidence are recovered in a timely and proper manner [47, 70]. It is pertinent to remember that DVI teams are always subject to the constraints and laws of the countries within which they are working and must adapt their investigation and identification procedures accordingly.

## Forensic anthropology education and DVI

In addition to their normal skillset of the recognition and interpretation of information from the skeleton, and a strong scientific understanding, there are a number of additional skills that are required of the forensic anthropologist who works as part of a DVI team. These include but are not limited to an understanding of the five phases of a DVI process; familiarity with the required documentation; an understanding of the legislation and hierarchy of the country where the DVI operation is being undertaken; and experience in the analysis of images including PMCT scans.

In recent years, there has been an increase in the number of degrees and postgraduate courses offered in forensic anthropology and there continues to be a large student demand for the subject [123, 124]. Many of these degrees, however, do not include training in mass fatality scenarios and do little to equip the newly qualified forensic anthropologist with the skills to be part of a DVI team. In addition to university courses, many countries have, therefore, tried to create a system whereby the experience and expertise of the forensic anthropologist is formally recognized. This is usually in the form of an accreditation or certification process overseen by a professional body. Currently, these quality controls vary from country to country and even where these are in place, compliance is not obligatory (See United States (http://theabfa.org/), Forensic Anthropology Society Europe (FASE) (http://www.forensicanthropology.eu/index.php/activities/fasecertification-process) and Royal Anthropological Institute, UK (https://www.therai.org.uk/forensicanthropology)). In each of these cases, however, certification processes have the advantage of providing the end user (i.e. the DVI team) with an indication of the experience and expertise of the forensic anthropologist. The process of certification provides access for the forensic anthropologist to mentoring and professional development, allowing them to progress as they gain experience [125]. Even where these systems exist, however, there are no requirements for forensic anthropologists to gain DVI experience working within a disaster mortuary environment. Many practitioners whether based at institutes of forensic medicine, medical examiners offices, or universities will only gain experience working independently or as part of a small team on domestic cases.

It is vital, therefore, that training opportunities in DVI are provided to forensic anthropologists in order to develop DVI preparedness. Raised awareness of the DVI process can be expanded in a number of ways including the provision of short courses within university degrees. Outside of these institutions practical training is also important to ensure an understanding of their role at the disaster mortuary or the scene [43, 45, 47, 95, 98, 126, 127]. In addition to understanding their own role, it is vital for forensic anthropologists to have an understanding of the roles of the other staff that they will be working alongside. Training exercises can assist with this and any local system of training and exercises should include all members of the DVI team [94, 128, 129]. Very important is the availability of time for honest reflection on lessons learnt from the exercise; the so-called “debrief” phase [130, 131].

Whilst exercises have their place, transparent reflection on past responses to mass fatality events can also play a part in this learning process and publications from practitioners add to the knowledge pool that is available to all practitioners [98, 132–137]. As outlined previously [34, 48], the forensic anthropologist may work with mortuary technicians, DNA scientists, and forensic pathologists in the auditing and review stages of the DVI. Other approaches can also help with training since the application of the DVI process is not restricted to mass fatality events. In some parts of the UK and Australia, the local DVI team (or personnel trained in DVI processes) is utilized in response to domestic events, which might not meet the criteria to be described as a “mass fatality” (see discussion above), but which involve fragmentation of the body/bodies. Including the forensic anthropologist in these cases ensures raised awareness of the different roles DVI members play as well as DVI processes and documentation.

Finally, in order to guarantee that the training of forensic anthropologists less experienced in DVI responses is fully supported by senior forensic anthropologists, each country should have a mentoring process in place that ensures that when a more experienced forensic anthropologist is deployed, their team, wherever possible, should include a forensic anthropologist who is still gaining DVI experience. This allows a transfer of knowledge and increases the potential pool of forensic anthropologists who are available when a disaster occurs [21].

## Concluding remarks

Having specialist knowledge of human anatomy and variability, forensic anthropologists are constantly considering new methods and techniques to augment human identification when preservation results in skeletonised or highly disrupted remains. Consequently, depending on the nature of the disaster, the inclusion of a forensic anthropologist in a DVI operation will substantially contribute to expediting identifications, as demonstrated by the strong role that forensic anthropologists have played in recent mass fatality events on a worldwide stage. The role of the forensic anthropologist in DVI will continue to evolve, depending on the recent and future developments in their own, and related, forensic disciplines. This paper has provided several examples of such developments and their effect on the DVI process.

The continuation of professional development in forensic anthropology is vital in order to continue to deliver highly skilled input in the future. The role that senior forensic anthropologists play in ensuring that they support training in the DVI process and provide professional mentoring to less experienced colleagues will ensure that this role remains one that continues to add value to the DVI team response.

The identification of the victims of a mass fatality event is a stressful and complex undertaking requiring the combined efforts of all members of a multidisciplinary team. It will not always be necessary to utilise the skills and expertise of a forensic anthropologist, but in many DVI operations they will prove to be a valuable asset.
